# Longitudinal multi-omics profiling of spinal muscular atrophy

**DOI:** 10.1016/j.neurot.2026.e00880

**Published:** 2026-03-12

**Authors:** Ivana Dabaj, Thi Hai Yen Nguyen, Emmanuelle Lagrue, Franklin Ducatez, Stéphane Allouche, Jérôme Ausseil, Andreea Seferian, Marta Gomez - Garcia de la Banda, Audrey Benezit, Aurélie Phelep, Mondher Chouchane, Stéphane Vasseur, Maud Chapart, Stéphane Marret, Susana Quijano Roy, Abdellah Tebani, Soumeya Bekri

**Affiliations:** a Normandie Univ, UNIROUEN, AIMS, SysMedLab, CHUROUEN, Department of Neonatalogy, Pediatric Intensive Care and Neuropediatrics, Referal Center for Neuromuscular Diseases, Referal Center for Lysosomal Diseases, 76000 Rouen, France; b Normandie Univ, UNIROUEN, AIMS, SysMedLab, CHUROUEN, Department of Metabolic Biochemistry, Referal Center for Lysosomal Diseases, Referal Center for Neuromuscular Diseases Nord-Est-Ile-de-France, 76000 Rouen, France; c Institut I-MOTION, Hôpital Armand Trousseau, Paris cedex 12, 75571, Paris, France; d Department of Biochemistry, University Hospital of Caen, Physiopathology and Imaging of Neurological Disorders, UMRS 1237, University of Caen Normandie, Caen, France; e Service de Biochimie, Institut Fédératif de Biologie, Centre Hospitalier Universitaire de Toulouse, Toulouse, France; f APHP Université Paris-Saclay, Pediatric Neuromuscular Unit, Hôpital Universitaire Raymond-Poincaré, Université de Versailles Saint-Quentin-en-Yvelines, Garches, France; g Department of Pediatric Neurology, French Competence Center for Neuromuscular Diseases, Dijon University Hospital Center, Hôpital d'Enfants, 14 rue Paul Gaffarel, 21079, Dijon, France; h MyoBank AFM-Institut de Myologie, Paris, France

**Keywords:** Spinal muscular atrophy, Metabolomics, Proteomics, Biomarkers, Systems biology, Nusinersen

## Abstract

Spinal muscular atrophy (SMA) is an autosomal recessive neuromuscular disorder caused by *SMN1* gene variants, leading to the degeneration of anterior horn cells in the spinal cord. It is a disabling disease with varying severity. Nusinersen, the first approved in France, has dramatically transformed SMA management. However, the significant variability in patient response and disease progression highlights a critical need for objective, measurable indicators. This study aims to identify biomarkers in cerebrospinal fluid (CSF) and plasma associated with the clinical status of treatment-naive patients and their disease progression during therapy. We performed targeted metabolomics and proteomics analyses on plasma and CSF samples from SMA patients before and after six months of treatment, along with controls. The differential analysis was carried out to discover the SMA biomarkers. We found that levels of acylcarnitines, biogenic amines, and neurology-related proteins were mainly elevated, while glycerophospholipids primarily decreased in SMA plasma samples compared to controls. The biomarkers showed good performance in distinguishing SMA from controls with plasma AUCs >0.9. NEFH and creatinine were among the most prominent biomarkers for SMA diagnosis. Besides, 26 neurology-related proteins were found to be altered in patient CSF compared to controls. Furthermore, 11 potential proteins were identified to distinguish patients with 2 copies of *SMN2* from those with 3 or 4 copies using plasma. By unveiling specific biomarkers, this study offers valuable insights for accurate disease diagnosis and monitoring treatment effectiveness. This enables personalized SMA management and accelerates the development of targeted therapies.

## Graphical abstract

Graphical abstract is created in https://BioRender.com.

**Figure:**
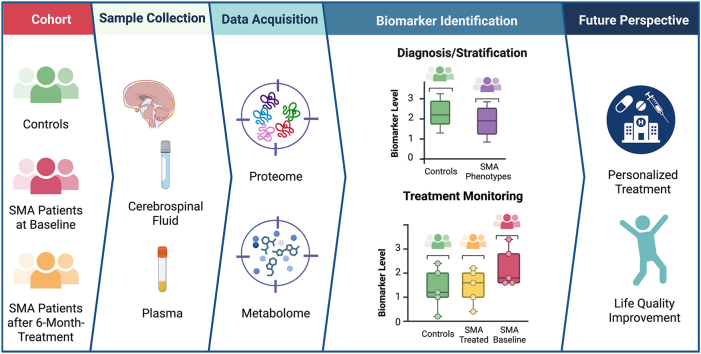

## Introduction

Spinal muscular atrophy (SMA) is an autosomal recessive neuromuscular disorder caused by pathogenic variants in the Survival Motor Neuron 1 (*SMN1*) gene, which encodes the SMN protein (survival motor neuron protein). This disease is characterized by progressive degeneration of the motor neurons, resulting in generalized muscular weakness and secondary moderate to severe impairment, with an incidence of 1:8400 births in Europe and 1:11,000 births in the USA [[1], [2], [3], [4], [5]]. Clinically, SMA exhibits a broad spectrum of severity and age of onset. The current classification differentiates five subtypes based on the age of onset, severity and maximal motor milestone reached: type 0 is the most severe, with death shortly after birth; type 1 is non-sitters, type 2 are sitters, type 3 are walkers and type 4 is adult onset [6,7]. Cognitive functions are usually preserved, even if in the most severely affected patients neurodegeneration could reach not only the anterior horn cells but also be widespread in the cerebral cortex, basal ganglia, brainstem, and cerebellum [8]. At the molecular level, homozygous deletions or loss-of-function variants in *SMN1* are the leading SMA-determining cause. The severity of the disease is modulated by the copy number of a nearly identical paralog gene, *SMN2* [4]. Both genes are located on chromosome 5q13. The difference between *SMN1* and *SMN2* lies in a few nucleotides, including one critical substitution in exon 7 of *SMN2* that leads to alternative splicing and exclusion of exon 7 in about 90 % of transcripts. This results in the production of an unstable SMN protein. Of note, *SMN2* is present in at least one copy, but the number of copies varies among individuals. Usually, the *SMN2* copy number is inversely correlated with SMA severity [4,6], but other factors, such as hypoxia and PLS3 expression [9], could influence the phenotype.

Over recent years, novel therapies have been developed to treat SMA. Nusinersen (Spinraza), a synthetic antisense oligonucleotide, binds to intron 7 of the *SMN2* pre-mRNA and promotes the production of a fully functional SMN protein [1,3,7,10]. It is administered intrathecally with a loading dose in the first two months (day 1, 15, 29, 64) and maintenance doses every fourth months [10]. The earlier the drug is given, the higher the success rate [6]. Other therapies are Onasemnogene Abeparvovec (Zolgensma®), a one-time *SMN1* gene replacement therapy given intravenously or intrathecally, and Risdiplam (Evrisdy®), an orally administered small molecule that modulates *SMN2* splicing [11]. The current innovative therapies can significantly alter the disease course. To facilitate presymptomatic diagnosis and improve outcomes, newborn screening programs for SMA have been implemented in several countries, including the USA, Germany, Belgium, and Australia. France is expected to launch its program in September 2025 [3,6]. Although *SMN2* copy number is the most significant known genetic modifier, the correlation between genotype and phenotype is incomplete [1,5,7,12,13]. Effective SMA management has markedly improved over the years through advances in understanding genotypes, phenotypes and severity which help in stratifying patients and in therapeutic decisions. Ongoing research would enable more personalized and more objective treatment plans.

Comprehensive omics approaches offer a powerful biological lens to understand disease mechanisms and treatment response. For instance, the SMN protein in blood or CSF has been used extensively as a biomarker for SMA. Treatments aim to raise their levels, with increases indicating target engagement and monitoring treatment response. However, their overlap across clinical subtypes limits their utility as severity markers [14]. Variability in disease severity, especially in milder or late-onset cases, makes decisions complex. Reliable biomarkers that predict severity and monitor response are essential. In this study, we focused on metabolomic and proteomic profiling of plasma and CSF samples from SMA patients before and after six months of Nusinersen treatment at two French centers. In this study, we performed metabolomic and proteomic profiling of plasma and CSF samples from SMA patients both longitudinally (before and after six months of Nusinersen treatment) and cross-sectionally (compared to control individuals). Our objectives were to identify biomarkers correlating with disease severity and monitoring treatment response. These findings could improve disease monitoring and support appropriate and timely treatment for SMA patients.

## Materials and Methods

### Patients and samples

Patients with a molecularly confirmed SMA diagnosis who underwent treatment with Nusinersen in two French centers were included. Plasma and CSF samples were collected during the regular visits for treatment (before treatment, day 15, day 30, day 64, day 184, every four months). Different scales to evaluate motor function (Motor Function Measurement, HINE, HFMSE, CHOP intend), respiratory function (vital capacity), and digestive and orthopedic statuses were used to evaluate the severity of the disease and efficacy of treatment. Fifty-three plasma samples SMA patients were selected for the study before treatment (baseline) and were compared to 71 age and sex matched plasma samples from controls (from two French centers: Caen University Hospital and Toulouse University Hospital). Twenty-three plasma samples at day 184 after treatment initiation were selected. Twenty-eight CSF samples before treatment and 22 CSF samples at day 184 after treatment initiation were analyzed. Age and sex matched CSF controls from 49 participants (Caen University Hospital and Toulouse University Hospital) have been used. All procedures were performed under the ethical standards of the institutional and/or national research committee and with the 1964 Helsinki Declaration and its later amendments or comparable ethical standards. To identify the biomarkers for SMA management, the comparisons between SMA and control groups were performed (i) SMA samples with different phenotype characteristics at baseline before Nusinersen treatment and control samples and (ii) SMA samples at baseline before Nusinersen treatment and samples at day 184 after treatment.

### Targeted metabolomics

#### Reagents

All reagents, internal and calibration standards, quality controls, test mix, and a patented 96-well filter plate required for the AbsoluteIDQ®p180 analysis are included in the kit or provided by Biocrates Life Science AG (Innsbruck, Austria). All metabolites were identified and quantified using isotopically labeled internal standards and multiple reaction monitoring as optimized and provided by Biocrates Life Sciences AG (Innsbruck, Austria).

#### Metabolite quantitation

Sample preparation was carried out according to the manufacturer's protocol [15,16]. Briefly, 10 μL of plasma was transferred to the upper 96-well plate and dried under a nitrogen stream. After that, 50 μL of a 5 % PITC solution was added to derivatize amino acids and biogenic amines. After incubation, the spots were dried again before the metabolites were extracted using 5 mM ammonium acetate in methanol (300 μL) into the lower 96-well plate for analysis after further dilution using the MS running solvent A. Quantification was carried out using isotopically labeled internal standards and a calibration curve [15,16]. The AbsoluteIDQ® p180 kit is a fully internal standards for quantitation. Amino acids and biogenic amines are determined in automated assay based on phenylisothiocyanate derivatization of the target analytes in bodily fluids using liquid chromatography-tandem mass-spectrometry LC-MS mode, acylcarnitines, phospholipids, sphingomyelins, and the sum of hexoses are analyzed in flow injection analysis. The analyses were performed following the instructions of the kit manufacturer using LC instrument Shimadzu UFLC System (Shimadzu, Prominence, Kyoto, Japan) coupled to the 4000 Qtrap mass spectrometer (Sciex, Framingham, MA, USA) with an electrospray ion source. Data acquisition and processing were performed using the Analyst 1.6.3 software (Sciex, Framingham, MA, USA).

### Targeted proteomics profiling

An amount of 1 μL of plasma and CSF samples were subjected to targeted proteomics by applying the multiplex proximity extension assay (PEA) technology (Olink Target 96) using Olink® Signature Q100. Protein measurement was conducted using the Olink Target 96 Neurology assay version v.3911 (Olink, Uppssala, Sweden), which includes 92 oligonucleotide-labeled antibody probe pairs targeting proteins related to neurobiological processes and neurological diseases. These proteins are associated with neurodegeneration and neuroinflammation, which are central to SMA pathology. Additionally, this panel includes promising markers of SMA, such as neurofilament light chain (NEFL) [17]. Once bound to the targets, new DNA amplicons, identified by barcodes corresponding to their respective antigens, were established. Measurement of the amplicons was conducted using the Fluidigm BioMark HD real-time PCR technique. The counts of known barcode sequences were converted into normalized protein expression (NPX) units, a relative protein quantification unit on a log2 scale. For the Olink data were processed using Olink's standard pipeline, which includes extension control, inter-plate control, and normalization to produce NPX values on a log2 scale. To monitor assay performance control samples were included on each plate, and inter-plate variation was assessed using the internal controls provided by Olink. Data was acquired with Olink Signature B100, and processed using NPX™ Signature software (v.1.9.0). NEFH in plasma and CSF was quantified using electrochemiluminescence assays (Meso Scale Discovery [MSD], Rockville, MD, USA). Data will be acquired using a SECTOR S 6000 plate reader (Meso Scale Diagnostics, Rockville, MD, USA). NEFH levels were then log-2 transformed and combined with NPX data for subsequent analysis.

### Phospholipase A2 activity assessment

Phospholipase A2 (PLA2) activity was measured in plasma and CSF based on the method from Fonteh et *al*. using Invitrogen EnzChek™ Phospholipase A2 Assay Kit (Thermo Fisher Scientific) [18]. To summarize, a Lipid Mix was created using 30 μL 10 mM dioleoylphosphatidylcholine, 30 μL 10 mM dioleoylphosphatidylglycerol, and 30 μL 1 mM PLA2 substrate. The substrate-liposome mix were generated by slowly adding 50 μL of Lipid Mix to 5 mL 1X PLA2 reaction buffer (50 mM Tris-HCl at pH 8.9, 100 mM NaCl, and 1 mM CaCl_2_) in a 20 mL beaker containing a small magnetic stir bar. The available diluted CSF and plasma samples were placed in 96-well plates, and PLA2 activity was activated by adding 50 μl of the substrate-liposome mix. Fluorescence was measured (excitation at 360 nm and emission at 460 nm), and the specific activity [relative fluorescent units (RFU)/μg protein/min] for each sample was calculated.

### Data visualization

All data analyses and visualizations were performed using R version 4.3.0. Significantly altered analytes were visualized as a volcano plot using the EnhancedVolcano R package (version 1.20.0) [19]. Upset plots were created to assess the commonality of the significant molecules between different comparisons. The UpSetR package (version 1.4.0) was used to generate the upset plots [20]. Heatmaps were conducted using the ComplexHeatmap R package (version 2.18.0) to visualize the level of molecules [21]. The *ggplot2* R package (version 3.January 5, 9000) was utilized to create violin plots [22].

### Data analysis

All data matrices are provided in supplementary tables. The data matrix was log2-transformed. Missing values were imputed using the nearest neighbor averaging algorithm using the KNNimp(.) function in the multiUS package version 1.2.3. The limma package was used for differential analysis, with sex and age considered by adding them as covariates. A mixed-effect model adjusting sex and age and treating sampleID as a random factor was performed for the comparison between samples collected after 184 days and at baseline using OlinkAnalyze package version 3.8.2 and lmerTest package version 3.1–3. Enrichment analysis was conducted on significant proteins using the Enrichr package version 3.2. Spearman correlation analysis was performed using the correlation package version 0.8.4. False discovery rates (FDR) were corrected using the Benjamini–Hochberg–Yekutieli method. Significantly altered molecules were determined with a cut-off of FDR 0.05. The diagnostic performance of significant molecules revealed from the comparison between the sample at baseline and the control was subsequently assessed. The potential biomarkers were evaluated based on the AUC through the classical univariate receiver operating characteristic (ROC) curve analyses in the MetaboAnalystR package version 4.0.0. The multivariate analyses consisting of three machine learning models of partial least squares discriminant analysis (PLS-DA), support vector machines (SVM), and random forest (RF) were implemented using the caret (version 6.0–94) package. The predictive models were validated by nested cross-validation. The process involved an outer loop for performance estimation and an inner loop for hyperparameter tuning. The dataset was partitioned into 5 stratified folds. For each iteration of the outer loop, a single fold was reserved as the test set to evaluate the final model's performance, while the remaining folds served as the training data for the inner loop, with a second level of 5-fold cross-validation performed to optimize model hyperparameters.

## Results

### Cohort overview

**Fig. 1:**
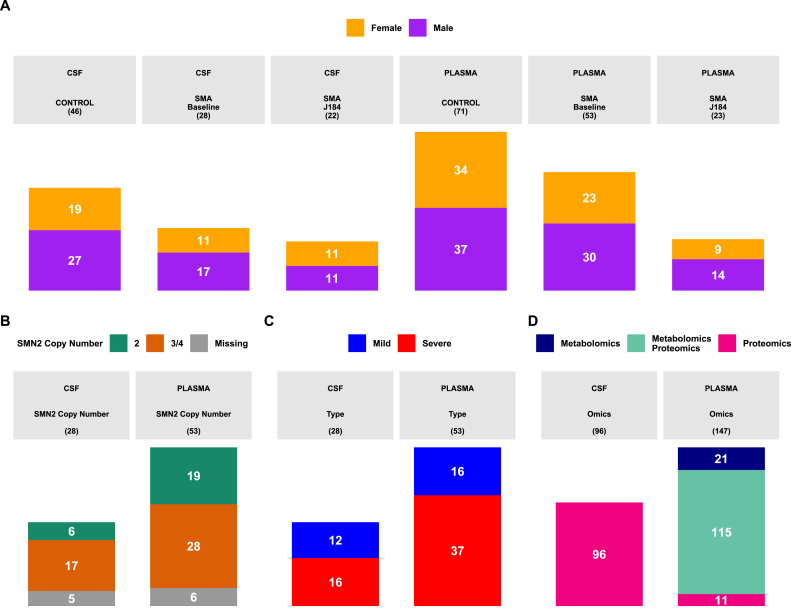
Overview of the cohort. a) Sex distribution in SMA patients and controls across matrices. b Stratification of patients according to *SMN2* Copy number in plasma and cerebrospinal fluid matrices (CSF). c) Cohort stratification regarding clinical severity. d) Overlapping samples between metabolomics and proteomics analysis.

This work aims to identify biomarkers of disease severity and follow-up using metabolomics and proteomics profiles in plasma along with metabolomics analysis in CSF samples across different groups: (i) SMA (Baseline) versus control samples; (ii) SMA (J184) (day 184 after treatment initiation) versus SMA (Baseline); (iii) SMA samples at baseline categorized by clinical severity: SMA (Severe) (type 1) versus controls samples and SMA (Mild) (types 2 and 3) versus control samples, SMA (Severe) versus SMA (Mild); (iv) SMA samples at baseline categorized by *SMN2*: SMN2 (Copy2) versus control samples, SMN2 (Copy3/4) (*SMN2* copy number ≥3) versus control samples, SMN2 (Copy3/4) versus SMN2 (Copy2). An overview of the cohort is presented in Fig. 1. The clinical characteristics of paired SMA samples after 6 months of treatment and at baseline are shown in Supplementary Table S1. Of note, patients after 6 months of treatment changed their ability to sit with/without support, eat alone, and take showers alone when compared to baseline status.

### Molecular perturbations in different SMA subgroups compared to controls

**Fig. 2:**
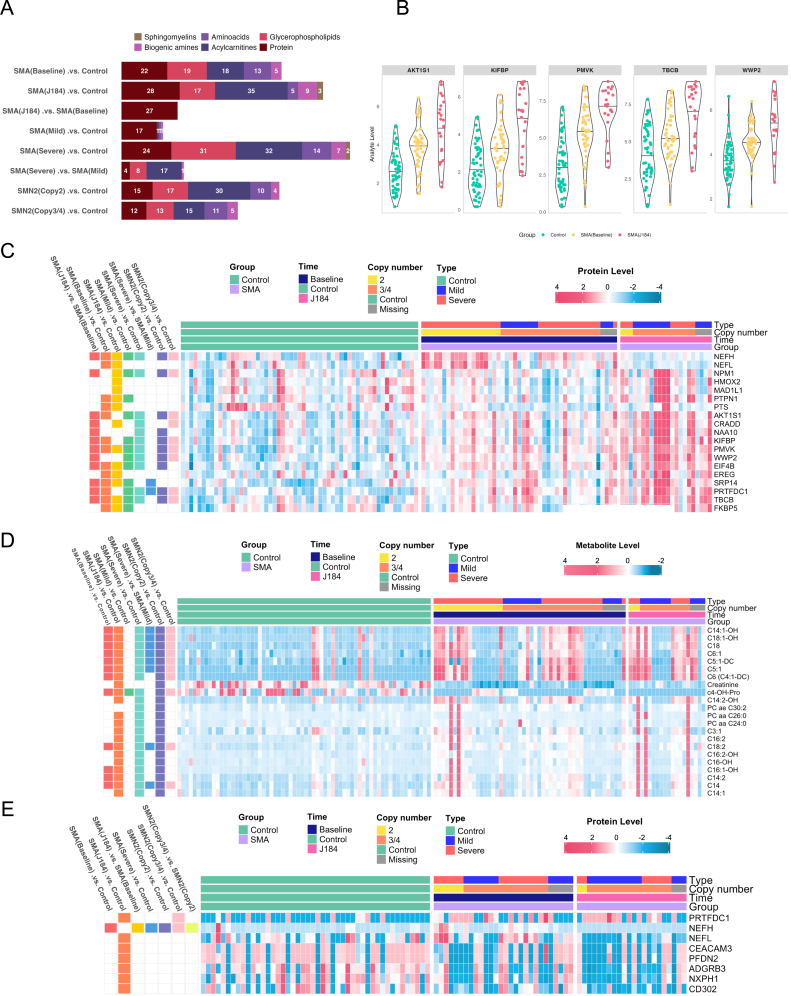
Differential analysis. a) Significantly altered molecules in SMA groups compared to controls using plasma samples (FDR <0.05). b) Altered neurology-related proteins in SMA compared to controls and in SMA during treatment using plasma samples. c) Heatmap of prominent neurology-related proteins in SMA groups compared to controls using plasma samples [FDR <0.05, log(FC) > 1.5]. D) Heatmap of prominent metabolites in SMA groups compared to controls using plasma samples [FDR <0.05, log(FC) > 1.5]. e) Heatmap of significantly altered neurology-related proteins in SMA groups compared to controls using cerebrospinal fluid samples [FDR <0.05, log(fold change) > 1].

For plasma samples, the statistical analysis identified 22 proteins and 55 metabolites (19 glycerophospholipids, 18 acylcarnitines, 13 amino acids and 5 biogenic amines) that were differentially altered in SMA (Baseline) compared to controls, while 28 proteins and 69 metabolites (35 acylcarnitines, 17 glycerophospholipids, 9 biogenic amines, 5 amino acids, and 3 sphingomyelins) were found in SMA (J184) compared to controls (Fig. 2a).

In terms of disease severity, the comparison between SMA (Mild) and SMA (Severe) with controls yielded 20 and 110 features, respectively. Moreover, 76 (15 proteins and 61 metabolites) and 56 (12 proteins and 44 metabolites) molecules were disturbed in *SMN2* (Copy2) and *SMN2* (Copy3/4) compared to controls. Among 19 prominent proteins with log(FC) > 1.5, 16 proteins (including WWWP2, EIF4B, PMVK, and NAA10) were up-regulated consistently from SMA (Baseline) to SMA (J184) compared to controls (Fig. 2b). Of note, PMVK expression level increased more than 4 times in SMA (Baseline) and more than 12 times in SMA (J184) compared to controls. Meanwhile, NEFH and NEFL expression were increased in SMA (Baseline), then decreased in SMA (J184) compared to controls. Conversely, PTS was down-regulated in SMA compared to control groups. Regarding metabolome, creatinine and c4-OH-Pro levels were lower, while acylcarnitines (compressing C18:1-OH, C18, C6:1, and C5:1), phosphatidylcholines (PCs) (PC aa C26:0, and PC aa C24:0), and ether-linked phosphatidylcholines [PC(O-)] PC ae C30:2 were higher in SMA than in control groups (Fig. 2c and d). Volcano plots resulting from differential analysis using plasma samples between SMA patients and controls are exhibited in Supplementary Figs. S1–S3. The top five significant analytes in each comparison are visualized in Supplementary Fig. S4.

Regarding CSF samples, 25 proteins were perturbed in SMA (J184) compared to controls (Fig. 2e, Supplementary Fig. S5). In terms of clinical severity, PRTFDC1 was altered in SMA (Severe) versus controls, as well as in SMA (Copy2) and SMA (Copy3/4) compared to controls. Among them, ADGRB3, NXPH1, and CD302 were down-regulated consistently in SMA compared to control groups. PRTFDC1 was up-regulated in SMA compared to the control groups. The whole data matrix with sample characteristics is presented in Supplementary Tables S2 and S3.

**Fig. 3:**
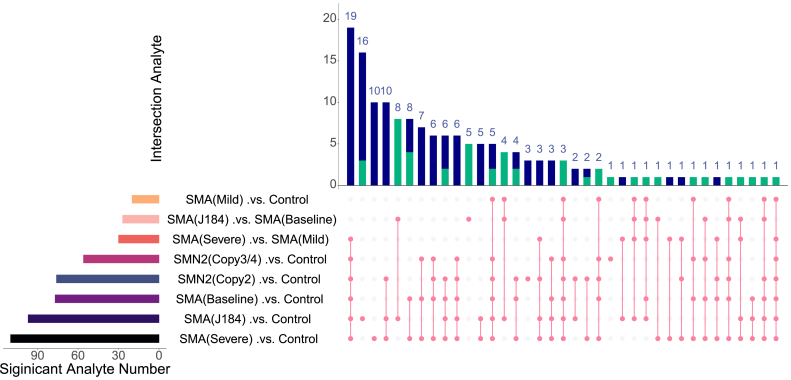
Upset plot of overlapped significant molecules in SMA groups compared to controls. A) Using plasma samples. B) Using cerebrospinal fluid samples.

The upset plot shows the intersection of significant omics features altered in the SMA groups compared to controls in the plasma analysis (Fig. 3). Significant molecules were highly overlapped between comparisons, revealing potential biomarkers for SMA groups with different phenotypes. PMVK, PRTFDC1, and AKT1S1 were shared among comparisons between SMA groups with different phenotypes versus controls. Significant proteins were then investigated to elucidate the underlying biological processes, revealing several altered pathways (Table 1). The complete list of significant molecules and their related statistics is presented in Supplementary Tables S4 and S5.

**Table 1:** Significant enrichment pathways related to spinal muscular atrophy.

Matrice	Comparison	Database	Term	Overlap	FDR	Neurology-related proteins
Plasma	SMA (baseline).vs. Control	GO:BP	Intermediate filament bundle assembly	2/7	6.02 x 10^−04^	NEFL, NEFH
Plasma	SMA (baseline).vs. Control	GO:BP	Supramolecular fiber organization	2/316	4.18 x 10^−02^	NEFL, TBCB
Plasma	SMA (Copy2).vs. Control	GO:BP	Intermediate filament bundle assembly	2/7	9.72 x 10^−04^	NEFL, NEFH
Plasma	SMA (Copy2).vs. Control	GO:BP	Negative regulation of protein phosphorylation	2/149	4.02 x 10^−02^	SFRP1, AKT1S1
Plasma	SMA (Copy2).vs. Control	GO:BP	Regulation of angiogenesis	2/205	4.02 x 10^−02^	SFRP1, PSG1
Plasma	SMA (Copy2).vs. Control	KEGG	mTOR signaling pathway	2/154	3.82 x 10^−02^	AKT1S1, EIF4B
Plasma	SMA (Copy3.4).vs. Control	KEGG	mTOR signaling pathway	2/154	1.69 x 10^−02^	AKT1S1, EIF4B
Plasma	SMA (J184).vs. Control	GO:BP	Negative regulation of insulin receptor signaling pathway	2/26	2.51 x 10^−02^	PTPN1, RPS6KB1
Plasma	SMA (J184).vs. Control	GO:BP	Negative regulation of cellular response to insulin stimulus	2/27	2.51 x 10^−02^	PTPN1, RPS6KB1
Plasma	SMA (J184).vs. Control	GO:BP	Regulation of protein transport	2/28	2.51 x 10^−02^	PTPN1, WWP2
Plasma	SMA (J184).vs. Control	GO:BP	Positive regulation of protein tyrosine kinase activity	2/38	3.48 x 10^−02^	PTPN1, EREG
Plasma	SMA (J184).vs. Control	GO:BP	Regulation of insulin receptor signaling pathway	2/44	3.74 x 10^−02^	PTPN1, RPS6KB1
Plasma	SMA (J184).vs. Control	KEGG	mTOR signaling pathway	3/154	1.34 x 10^−02^	RPS6KB1, AKT1S1, EIF4B
Plasma	SMA (J184).vs. Control	KEGG	ErbB signaling pathway	2/85	2.79 x 10^−02^	RPS6KB1, EREG
Plasma	SMA (J184).vs. Control	KEGG	Colorectal cancer	2/86	2.79 x 10^−02^	RPS6KB1, EREG
Plasma	SMA (J184).vs. Control	KEGG	PI3K-Akt signaling pathway	3/354	2.79 x 10^−02^	RPS6KB1, EIF4B, EREG
Plasma	SMA (J184).vs. Control	KEGG	Longevity regulating pathway	2/102	2.79 x 10^−02^	RPS6KB1, AKT1S1
Plasma	SMA (J184).vs. Control	KEGG	Insulin resistance	2/108	2.79 x 10^−02^	PTPN1, RPS6KB1
Plasma	SMA (J184).vs. Control	KEGG	AMPK signaling pathway	2/120	2.93 x 10-^02^	RPS6KB1, AKT1S1
Plasma	SMA (J184).vs. Control	KEGG	Insulin signaling pathway	2/137	2.95 x 10^−02^	PTPN1, RPS6KB1
Plasma	SMA (J184).vs. Control	KEGG	Autophagy	2/137	2.95 x 10^−02^	RPS6KB1, AKT1S1
Plasma	SMA (J184).vs. SMA (baseline)	GO:BP	Negative regulation of protein kinase activity	3/88	2.74 x 10^−02^	PTPN1, NPM1, AKT1S1
Plasma	SMA (J184).vs. SMA (baseline)	GO:BP	Protein dephosphorylation	3/124	2.74 x 10^−02^	PTPN1, PPP3R1, ILKAP
Plasma	SMA (J184).vs. SMA (baseline)	GO:BP	Negative regulation of insulin receptor signaling pathway	2/26	2.74 x 10^−02^	PTPN1, RPS6KB1
Plasma	SMA (J184).vs. SMA (baseline)	GO:BP	Negative regulation of cellular response to insulin stimulus	2/27	2.74 x 10^−02^	PTPN1, RPS6KB1
Plasma	SMA (J184).vs. SMA (baseline)	GO:BP	Regulation of protein transport	2/28	2.74 x 10^−02^	PTPN1, WWP2
Plasma	SMA (J184).vs. SMA (baseline)	GO:BP	Dephosphorylation	3/147	2.87 x 10^−02^	PTPN1, PPP3R1, ILKAP
Plasma	SMA (J184).vs. SMA (baseline)	GO:BP	Positive regulation of protein tyrosine kinase activity	2/38	3.61 x 10^−02^	PTPN1, EREG
Plasma	SMA (J184).vs. SMA (baseline)	GO:BP	Regulation of insulin receptor signaling pathway	2/44	4.24 x 10^−02^	PTPN1, RPS6KB1
Plasma	SMA (J184).vs. SMA (baseline)	KEGG	mTOR signaling pathway	3/154	4.83 x 10^−02^	RPS6KB1, AKT1S1, ATP6V1F
Plasma	SMA (severe).vs. Control	GO:BP	Intermediate filament bundle assembly	2/7	2.87 x 10^−04^	NEFL, NEFH
Plasma	SMA (severe).vs. Control	KEGG	mTOR signaling pathway	2/154	3.07 x 10^−02^	AKT1S1, EIF4B
Plasma	SMA (severe).vs. Control	KEGG	Amyotrophic lateral sclerosis	2/364	4.37 x 10^−02^	NEFL, NEFH

Abbreviation: SMA, Spinal muscular atrophy.

### Diagnosis biomarker

Univariate ROC curve analysis using plasma samples from SMA patients at baseline and controls yielded AUCs >0.6 of 12 proteins (Supplementary Table S6). Of note, PMVK, EIF4B and PRTFDC1 exhibited AUCs ≥0.8. For plasma metabolomics, there were 11 significant metabolites with AUCs >0.6. Among them, 6 metabolites had AUCs from 0.70 to 0.78, and C18:2 had an AUC of 0.83 (Supplementary Table S7). Creatinine had a good diagnostic ability with AUC = 0.94.

**Fig. 4:**
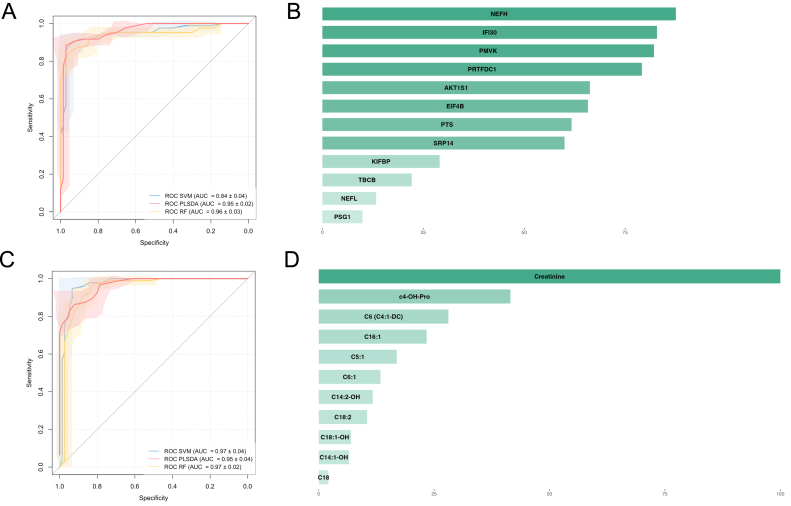
Diagnosis biomarker validation. a) Receiver operating characteristic (ROC) curves from classification models using significant neurology-related proteins from plasma samples. b) The mean importance score of significant neurology-related proteins in plasma samples classification models. c) ROC curves from classification models using significant metabolites from plasma samples. d) The mean importance score of significant metabolites in plasma samples classification models. PLS-DA: partial least squares discriminant analysis; SVM: support vector machines; RF: random forest; ROC: receiver operating characteristic curve.

The biomarkers, both proteins and metabolites, showed good performance (almost AUCs >0.9) in classifying the plasma samples from SMA with control groups (Fig. 4). Three models, including RF, SVM, and PLS-DA, demonstrated AUCs of 0.96 ± 0.03, 0.84 ± 0.04, and 0.95 ± 0.02 using protein markers (Fig. 4a). NEFH, IFI30, and PMVK had the most important roles in the classifications (Fig. 4b). And AUCs of 0.97 ± 0.02, 0.93 ± 0.04, and 0.91 ± 0.04 using metabolite marker applied RF, SVM, and PLS-DA algorithms with the prominent contribution of creatinine and *cis*-4-Hydroxyproline (C4 OH Pro) (Fig. 4c and d).

### The alteration of correlation with NEFH and NEFL across time

**Fig. 5:**
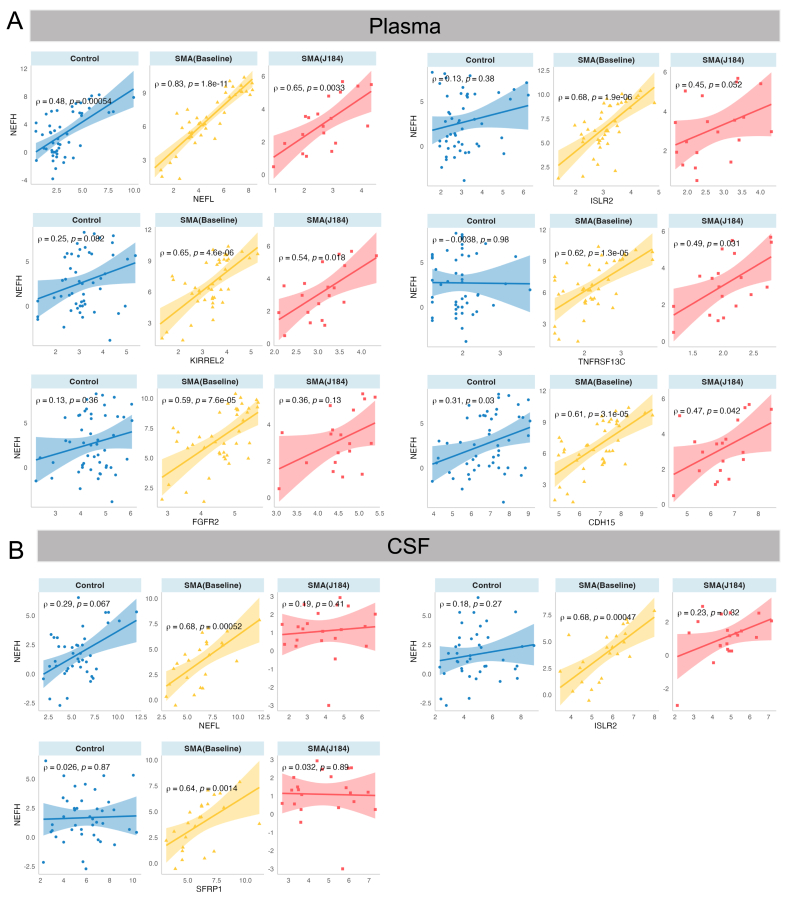
Alterations of significant correlations between NEFH and other molecules in the baseline SMA group. a) Plasma samples. b) Cerebrospinal fluid (CSF) samples. CSF: cerebrospinal fluid.

Since NEFH and NEFL were reported to be potential biomarkers for SMA, the correlations between NEFH and NEFL with other molecules in baseline SMA samples were investigated. The significant correlations are shown in Supplementary Table S8-10*.* Remarkably, NEFH was significantly up-regulated in SMA (Baseline) compared to controls. Therefore, we focused on the alterations of these significant correlations of NEFH in SMA (Baseline) compared to SMA (J184) and controls. This protein was positively correlated with 6 proteins (NEFL, ISLR2, KIRREL2, TNFRSF13C, FGFR2, and CDH15) in plasma baseline samples (Fig. 5a). The correlations increased in the baseline compared to controls and decreased in the samples after 184 days of treatment. For CSF samples, significant correlations were observed between NEFH with 3 proteins (NEFL, ISLR2, and SFRP1) at baseline (Fig. 5b). Interestingly, NEFH was correlated with NEFL and ISLR2 regardless of sample type, whether plasma or CSF. In the same vein, the positive correlations between NEFH and NEFL, ISLR2, and SFRP1 were augmented in baseline compared to controls and declined after 184 days of treatment.

### Potential protein biomarker for stratification of patients with different SMN2 copy numbers

**Fig. 6:**
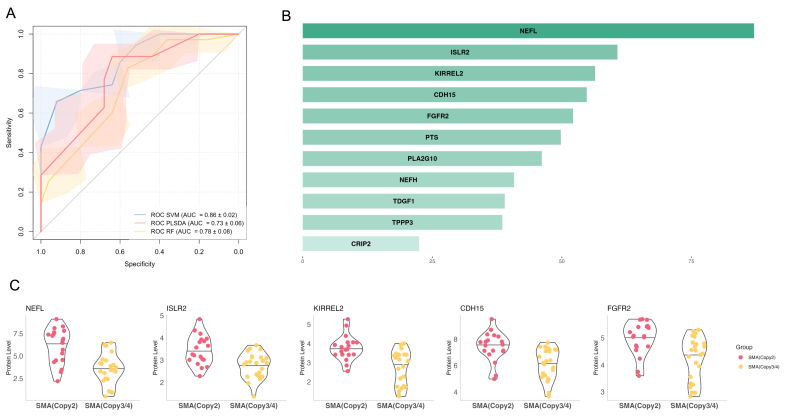
Classification of patients with different *SMN2* copy numbers. a) Receiver operating characteristic curve curves from classification models using selected neurology-related proteins from plasma samples. b) The mean importance score of selected neurology-related proteins in plasma samples classification models. c) Violin plots of the top 5 potential neurology-related proteins. PLS-DA: partial least squares discriminant analysis; SVM: support vector machines; RF: random forest; ROC: Receiver operating characteristic curve.

In our cohort, machine learning approaches using all analytes showed that plasma proteome had a differentiated capacity, while plasma metabolome and CSF profiles failed to classify SMA (Copy2) with SMA (Copy3/4) groups. Among the top 20 important proteins, 11 overlapped proteins from RF, SVM, and PLS-DA models were selected to be validated subsequently. Fig. 6a exhibited acceptable performance with AUCs of 0.86 ± 0.02, 0.73 ± 0.06, and 0.76 ± 0.08 using SVM, PLS-DA, and RF algorithms. NEFL, ISLR2, KIRREL2, CDH15, and FGFR2 contributed the most to the classification of patients with different *SMN2* copy numbers (Fig. 6b). All these 5 proteins were upregulated in SMA (Copy2) compared to SMA (Copy3/4) groups (Fig. 6c).

### Phospholipase A2 activity as a potential biomarker for response to treatment

**Fig. 7:**
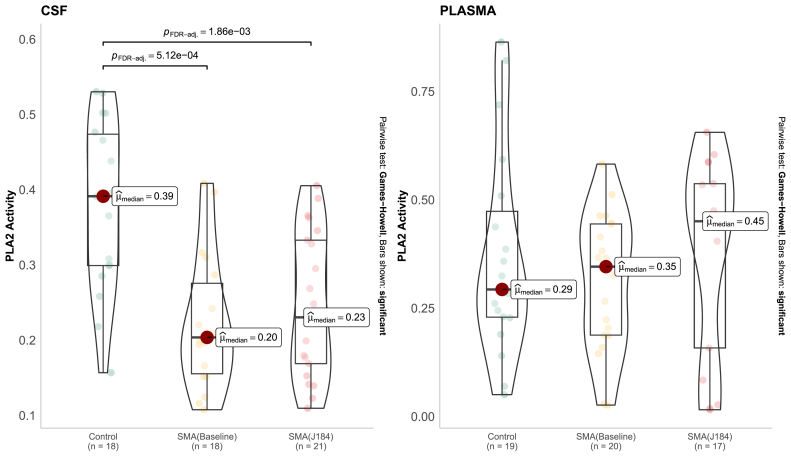
Phospholipase A activity in the plasma and cerebrospinal fluid (CSF).

In SMA plasma samples, PC species levels are mainly elevated, whereas lysoPC levels are generally reduced compared to controls (Table S4). Phospholipase A2 catalyzes the deacylation of PC into LysoPC [23]. Consequently, our findings may indicate diminished phospholipase A2 activity in SMA patients. To verify this, we evaluated phospholipase A2 activity. As a result, PLA2 activity in CSF significantly declined in SMA samples at baseline and after 6 months of treatment compared to controls (Fig. 7). However, no significant changes in PLA2 activity were observed in plasma samples, although there was an increasing trend over time.

## Discussion

SMA disease is a neuromuscular disorder characterized by clinical heterogeneity. Currently, there is a pressing need to identify reliable biomarkers for patient stratification and disease monitoring. The emergence of new targeted and innovative therapies highlights the importance of developing tools for early diagnosis and treatment assessment. These specific biomarkers could streamline patient stratification. We performed targeted metabolomic and proteomic analyses on an SMA cohort before and after Nusinersen treatment, comparing them to control individuals. The study revealed significant metabolic and proteomic remodeling in untreated SMA patients. We observed differential changes in these profiles when comparing SMA patients to controls, as well as among SMA individuals with varying clinical severities and copy numbers.

Several prominent proteins were revealed for SMA diagnostic. Neurofilaments, phosphorylated neurofilament heavy chain (pNEFH) and NEFL, are released during axonal degeneration. Their levels increase in CSF and plasma/serum of patients with motor neuronopathy and are associated with disease activity [1,7,14]. In this work, plasma NEFH was significantly upregulated in SMA patients at baseline compared to controls and then decreased after 184 days of Nusinersen treatment. This aligns with previous studies showing higher levels at baseline in SMA versus non-SMA and a rapid decline at 2 months of treatment, which was more pronounced at 10 months and varied according to clinical severity [10,24]. NEFH is a neuronal cytoskeleton protein released into circulation after axonal damage, indicating neuronal degeneration, and its high levels are found in neurodegenerative diseases [24,25].

The molecular alterations observed in this study likely reflect a combination of primary effects of SMN deficiency and secondary downstream consequences of motor neuron degeneration, muscle wasting, and systemic metabolic adaptation. Changes in neurofilaments and axon-related proteins detected in both plasma and CSF and partially reversed after Nusinersen treatment support a direct link to SMN-dependent neuronal pathology. In contrast, alterations in metabolites such as creatinine, acylcarnitines, and phospholipids are more consistent with secondary effects related to denervation, reduced muscle mass, and altered energy metabolism.

Moreover, NEFH was positively correlated in plasma and CSF with NEFL and ISLR2 (Immunoglobulin superfamily containing leucine-rich repeat protein 2). The correlation between neurofilament heavy and light chains has been described, and both are considered potential biomarkers [10,14,26,27]. NEFL shows a strong correlation with disease severity, being higher in patients with 2 *SMN2* copies compared to those with 3 and 4 copies. It is also significantly elevated in patients at baseline versus controls, aligning with reported works [28,29]. Moreover, younger patients showed a greater reduction in NEFL levels compared to older patients, therefore predicting motor improvement following treatment with Nusinersen [30]. Besides, ISLR2 is a protein involved in axon guidance in brain development, thought to positively regulate axon extension and help reorganize the cytoskeleton of developing neurons [31]. The strong correlation between ISLR2 and neurofilaments in plasma and CSF may be due to their roles in axon development and stability. We observed a significant negative correlation between NEFH and four other plasma proteins (KIRREL2, TNFRSF13C, FGFR2, and CDH15) with severity, as their levels were higher in patients with 2 *SMN2* copies compared to those with 3 or 4 copies. Our predictive models identified another potential plasma diagnostic biomarker, IFI30, or Lysosomal Thiol Reductase, which helps unfold proteins for lysosomal breakdown and signals. IFI30 was decreased in patients at baseline compared to controls. One study suggested it might predict treatment response, as Nusinersen responders showed less decrease in CSF levels of IFI30 (along with ARSB, ENTPD2, and NEFL) than non-responders [30]. Moreover, high expression of PMVK (Phosphomevalonate kinase) was observed in the plasma of SMA patients at baseline compared to controls, and after treatment compared to before treatment, making it a prominent potential biomarker. PMVK is an ATP-dependent, peroxisomal enzyme that converts mevalonate-5-phosphate into mevalonate-5-pyrophosphate in the cholesterol biosynthesis pathway and the isoprenoid synthesis pathway. Intronic variants in the PMVK gene have been associated with the wearing-off phenomenon in Parkinson's disease, a neurodegenerative disease [32]. Besides, we determined several novel CSF potential biomarkers such as ADGRB3 (Adhesion G protein-coupled receptor B3), PRTFDC1 (Phosphoribosyltransferase domain-containing protein 1), CEACAM3 (a member of the family of carcinoembryonic antigen-related cell adhesion molecules), PFDN2 (Prefoldin subunit 2), CD302 (C-type lectin domain family 13 member A), NXPH1 (Neurexophilin-1) that downregulated in SMA patients after 6 months of treatment. External validation of the novel potential biomarkers is warranted in future work using an independent cohort.

Further potential response markers were described in the literature were response to treatment was found correlated to an increase in OL1A2 and GRIA4 proteins, reduction in SEMA6A in CSF of SMA type III [33,34] while in SMA type I an increase in Apolipoproteins (APOA1, APOE) and Transthyretin (TTR). Moreover, an increase in PARK7 (DJ-1) with decrease in Cathepsin D (CTSD) were found in CSF of responders [35].

Metabolome investigations were also SMA informative and provide insights into the fundamental mechanisms of the disease. Of note, plasma creatinine was found to be a remarkable biomarker for SMA diagnosis. Serum creatinine has been related to muscle deterioration, but its variability is broad. The increase in creatinine with decreasing creatine kinase during Nusinersen treatment suggests reduced muscle wasting and better energy metabolism. However, further prospective studies are necessary [36,37]. Metabolic abnormalities (alteration of fatty acid metabolism, glucose tolerance impairment, and mitochondrial defects) were reported in SMA animals and patients, and there is a possible impact of SMN deficiency on metabolic abnormalities [38,39].

Recent metabolomics studies highlight predictive and diagnostic metabolites for SMA subtypes stratification and to predict nusinersen efficacy (N-myristoyl arginine, Cinobufagin, 4-chlorophenylacetic acid, methylthioadenosine and methyladenosine, creatinine) [34,[40], [41], [42], [43]] As in our study, creatinine was found to be lower in SMA patients and inversely correlated with disease severity [35].

Acylcarnitines (C2, C5:1, and C16:1 in plasma), intermediate products of fatty acid oxidation, were identified to be elevated in SMA samples compared to controls. Several reports mentioned dyslipidemia with increased cardiovascular risk factors, liver steatosis, elevated concentrations of acylcarnitines with a decrease in free plasma carnitine [44]. The deregulation of fatty acid metabolism may be due to denervation, which results in loss of muscle fiber function and, thus, a decrease in the flux of beta-oxidation [45]. Notably, the plasma metabolome did not distinguish SMA patients at baseline from those after six months of treatment, potentially due to the lower signal-to-noise ratio of the metabolome and the shorter time window, which may be insufficient to observe the treatment effect. Additionally, we observed that PC species levels were primarily increased, while lysoPC levels tended to decrease in SMA plasma samples compared to controls. Moreover, PLA2 activity in CSF significantly declined in SMA samples at baseline and after 6 months of treatment compared to controls. Dysregulation of PLA2 activity or changes in PC/lysoPC levels might affect membrane integrity and signaling pathways that connect receptor agonists, oxidative agents, and proinflammatory cytokines to arachidonic acid release [46]. PLA2 enzymes, through their role in lipid metabolism, are central players in the complex pathology, including neuroinflammation, neurotransmission, and apoptosis of various neurodegenerative disorders such as Amyotrophic Lateral Sclerosis (ALS) and Alzheimer's disease [47,48], where PLA2-driven lipid remodeling contributes to neuroinflammation, oxidative stress, and mitochondrial dysfunction. In ALS, lipidomic shifts involving PCs have been identified in plasma and linked to disease progression. Furthermore, the shift from PC to lysoPC reflects alterations in cell membrane turnover and degradation. The presence of this imbalance in our SMA cohort suggests that, despite the specific genetic cause of SMA, the downstream metabolic consequences of motor neuron degeneration may share common pathways with other motor neuron diseases. More confirmation is needed, such as checking for consistency in test results, ruling out any influence from blood contamination, and looking into how PC/lysoPC ratios relate to support these observations.

Furthermore, we compared the metabolomics and proteomics profiles of controls and SMA patients according to the SMA clinical phenotype (severe: type 1 and moderate types 2/3) and according to the copy number of the *SMN2* gene. This may help better stratify SMA patients than clinical-based strategies. Among proteins that differentiate patients with two SMN2 copies from those with three or four copies, TNFRSF13C, Tumor Necrosis Factor Receptor Superfamily Member 13C, also known as BAFF receptor, plays a role in B-cell survival. Recent research indicates that immune dysregulation and neuroinflammation may contribute to the pathogenesis of SMA [49]. Therefore, alterations in BAFF signaling leading to an increase in TNFRSF13C could alter disease progression or response to treatment. FGFR2 (Fibroblast Growth Factor Receptor 2) plays a crucial role in neurogenesis, neuronal survival, and synaptic plasticity [50]. Our findings indicate that FGFR2 shows a reduction with more severe phenotypes and could be a valuable biomarker for disease severity and progression. Altered FGFR pathways are also observed in neurodegenerative diseases such as Alzheimer's disease [51]. FGF2 ligand has been studied for its neuroprotective effects and potential modulation of motor neurons. This may be due to its role in regulating satellite cells, thus implicating it in muscle repair and regeneration. Additionally, FGFR-mediated pathways influence the entire motor unit, from axon outgrowth to the maintenance of synapses and muscle regeneration [50]. Recent studies stratify patient using omics profiles and associate abnormalities in glucose metabolism with SMA type I, coagulation processes with SMA type II, complement cascade activation and amino acide metabolism with SMA type III and axonogenesis as well as axon development abnormalities with all subtypes [34,35].

The observed discordance between PLA2 activity in plasma and CSF might highlight its compartment-specific nature. While plasma PLA2 levels exhibited no significant changes over time, they showed higher baseline levels in patients compared to controls, potentially reflecting muscle mass loss. In contrast, CSF PLA2 activity was significantly decreased in patients both at baseline and after six months of treatment, indicating a link to altered lipid metabolism in motor neurons. This suggests that PLA2 activity serves as a distinct metabolic marker within the central nervous system and peripheral tissues, rather than a uniform systemic indicator.

While *SMN2* copy number and clinical assessments remain key tools, they do not fully capture the variability in disease severity. The molecular markers identified here may provide additional information that could help distinguish patients with similar SMN2 copy numbers or track biological changes over time. They can empower existing assessments, and could potentially contribute to more refined patient stratification and monitoring in future studies.

This study has several limitations. First, its retrospective design resulted in missing data for demographics, motor and respiratory assessments, and electrophysiological measurements, restricting longitudinal analyses to only baseline and six months. This could also influence the confounding effect of the clinical variables. Second, some clinical benefits of treatment may only emerge after 12–18 months, so the six-month follow-up may not fully capture the treatment's effects or identify all relevant biomarkers. Third, certain subgroups were small, limiting the robustness and statistical significance of some findings; larger prospective studies are needed to validate these results. Additionally, pre-analytical information (e.g., fasting status, sample collection, processing, and freeze–thaw cycles) was inconsistently available, potentially introducing variability. Finally, missing data were handled using KNN imputation across platforms to preserve multivariate covariance, but this approach does not explicitly account for below-LOD values and may introduce analytical bias. Future studies should explore LOD-aware imputation methods and validate findings in independent SMA cohorts.

Studying rare diseases like SMA limited the availability of certain sample types, such as specific CSF samples and complete pre/post-treatment pairs. However, our robust longitudinal multi-omics approach, including plasma and CSF data, yielded comprehensive molecular insights into SMA pathophysiology and treatment response. By exploring the complex molecular landscape of SMA, our study revealed three key findings: first, distinct proteomic and metabolomic profiles in plasma and CSF that differentiate SMA patients at baseline from controls; second, molecular signatures that distinguish clinical subtypes; and third, through longitudinal analyses, molecular changes associated with treatment response. Several of these biomarkers showed potential diagnostic value and correlations, which could support earlier detection, more precise patient stratification, and objective monitoring of treatment response through accessible blood tests, ultimately improving patient management.

With the increasing implementation of neonatal SMA screening, there is a growing need to better characterize intermediate phenotypes. Although SMN2 copy number is an important prognostic factor, it does not fully account for the variability in clinical outcomes. The identification and validation of additional biomarkers will therefore be crucial to refine phenotyping and guide early therapeutic decisions.

## Author contributions

ID, AT and SB conceived and designed the study. SV and MC provided patients samples. JA, SA provided samples of controls. AP, EL, AS, MC provided the clinical data of one center; ID, AB, MGGB, SQR provided the clinical data of the other center. NTHY, FD, and AT performed statistical analyses, data visualization, illustrations, and data interpretation. ID, NTHY, and SB wrote the original draft. SB and AT edited the final manuscript. All authors read and approved the manuscript.

## Declaration of competing interest

The authors declare that they have no known competing financial interests or personal relationships that could have appeared to influence the work reported in this paper.
