# Supplementary material for: Longitudinal multi-omics profiling of spinal muscular atrophy
Source: Neurotherapeutics. 2026 Mar 12;23(2):e00880. doi: 10.1016/j.neurot.2026.e00880 (PMC12996660; doi:10.1016/j.neurot.2026.e00880)

**Supplemntary material**

**Longitudinal multi-omics profiling of spinal muscular atrophy**

Ivana Dabaj ^1,2^, Nguyen Thi Hai Yen ^2^, Emmanuelle Lagrue ^3^, Franklin Ducatez ^1,2^, Stéphane Allouche ^4^, Jérôme Ausseil ^5^, Andreea Seferian ^3^, Marta Gomez - Garcia de la Banda ^6^, Audrey Benezit ^6^, Aurélie Phelep ^3^, Mondher Chouchane ^7^, Stéphane Vasseur ^8^, Maud Chapart ^8^, Stéphane Marret ^1,2^, Susana Quijano Roy ^6^, Abdellah Tebani ^2*^, Soumeya Bekri ^2^

^1^ Normandie Univ, UNIROUEN, AIMS, SysMedLab, CHUROUEN, Department of Neonatalogy, Pediatric Intensive Care and Neuropediatrics, Referal Center for Neuromuscular Diseases, Referal Center for Lysosomal Diseases, 76000 Rouen, France ; ivana.dabaj@chu-rouen.fr; franklin.ducatez@chu-rouen.fr; stephane.marret@chu-rouen.fr

^2^ Normandie Univ, UNIROUEN, AIMS, SysMedLab, CHUROUEN, Department of Metabolic Biochemistry, Referal Center for Lysosomal Diseases, Referal Center for Neuromuscular Diseases Nord-Est-Ile-de-France, 76000 Rouen, France ; ivana.dabaj@chu-rouen.fr; thi-hai-yen.nguyen@univ-rouen.fr; franklin.ducatez@chu-rouen.fr; [abdellah.tebani@chu-rouen.fr](mailto:abdellah.tebani@chu-rouen.fr); [soumeya.bekri@chu-rouen.fr](mailto:Soumeya.bekri@chu-rouen.fr)

^3^ Institut I-MOTION, Hôpital Armand Trousseau, Paris cedex 12, 75571, Paris, France ; [a.seferian@institut-myologie.org](mailto:a.seferian@institut-myologie.org); a.phelep@institut-myologie.org; lagrue@univ-tours.fr

4 Department of Biochemistry, University Hospital of Caen, Caen, France, Physiopathology and Imaging of Neurological Disorders, UMRS 1237, University of Caen Normandie, Caen, France.

5 Service de Biochimie, Institut Fédératif de Biologie, Centre Hospitalier Universitaire de Toulouse, Toulouse, France; [jerome.ausseil@inserm.fr](mailto:jerome.ausseil@inserm.fr)

6 APHP Université Paris-Saclay, Pediatric Neuromuscular Unit, Hôpital Universitaire Raymond-Poincaré, Université de Versailles Saint-Quentin-en-Yvelines, Garches, France; audrey.benezit@aphp.fr; susana.quijano-roy@aphp.fr [marta.gomezgarciadelabanda@aphp.fr](mailto:marta.gomezgarciadelabanda@aphp.fr)

^7^ Department of Pediatric Neurology, French Competence Center for Neuromuscular Diseases, Dijon University Hospital Center, Hôpital d'Enfants, 14 rue Paul Gaffarel, 21079, Dijon, France; [mondher.chouchane@chu-dijon.fr](mailto:mondher.chouchane@chu-dijon.fr)

8 MyoBank AFM-Institut de Myologie, Paris, France ; [s.vasseur@institut-myologie.org](mailto:s.vasseur@institut-myologie.org); m.chapart@institut-myologie.org

* Correspondence:

Prof. Abdellah TEBANI

Department of Metabolic Biochemistry,

AIMS, Systems Medicine Lab,

Referal Center for Lysosomal Diseases,

Referal Center for Neuromuscular Diseases Nord-Est-Ile-de-France,

Rouen University Hospital,

F-76000 Rouen, France

abdellah.tebani@chu-rouen.fr

Figure S1. Volcano plot resulting from differential analysis using plasma samples between A. SMA(Baseline) vs control. B. SMA(J184) vs control. C. SMA(J184) vs SMA(Baseline).


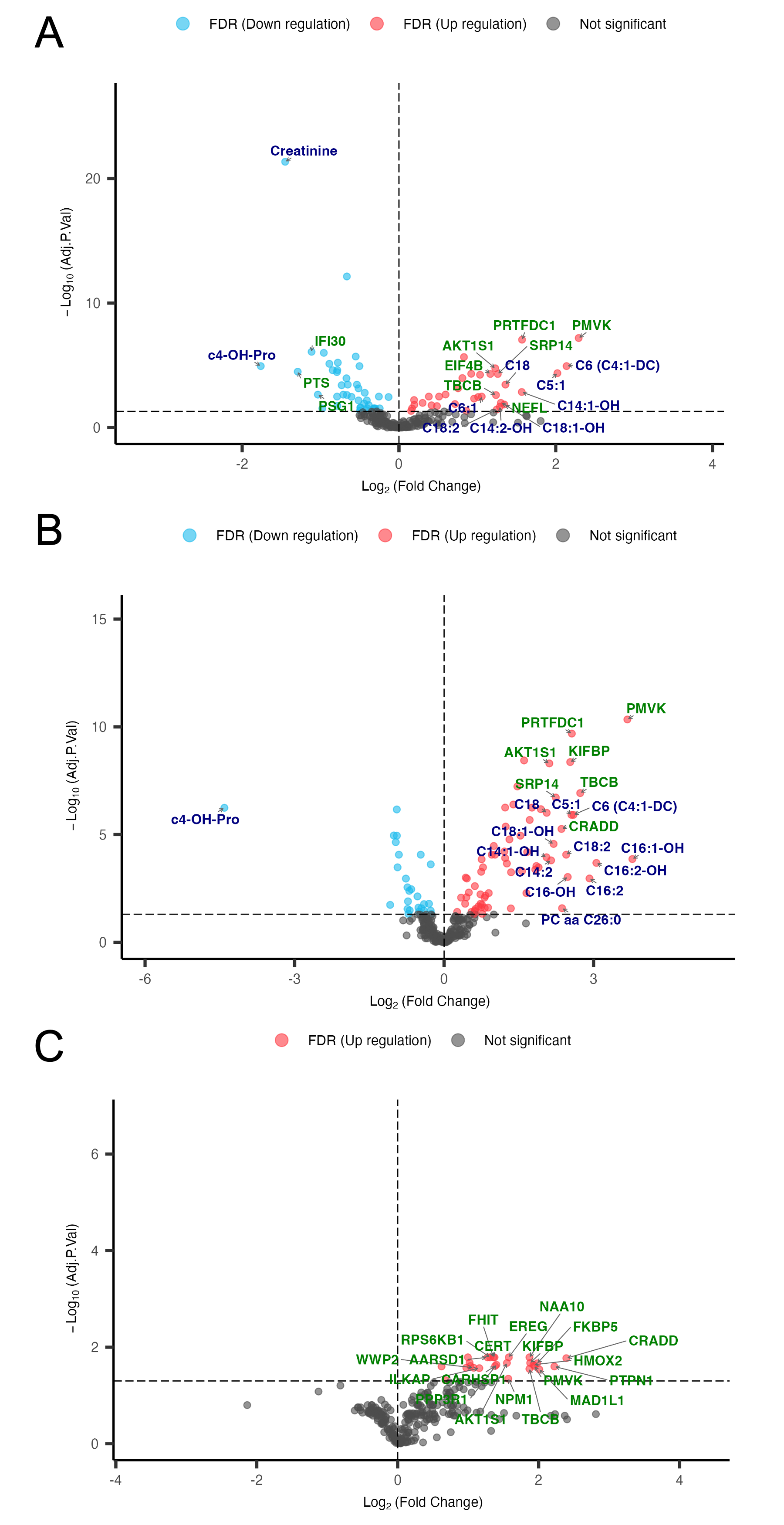


Figure S2. Volcano plot resulting from differential analysis using plasma samples between A. SMA(Mild) vs control. B. SMA(Severe) vs control. C. SMA(Severe) vs SMA(Mild).


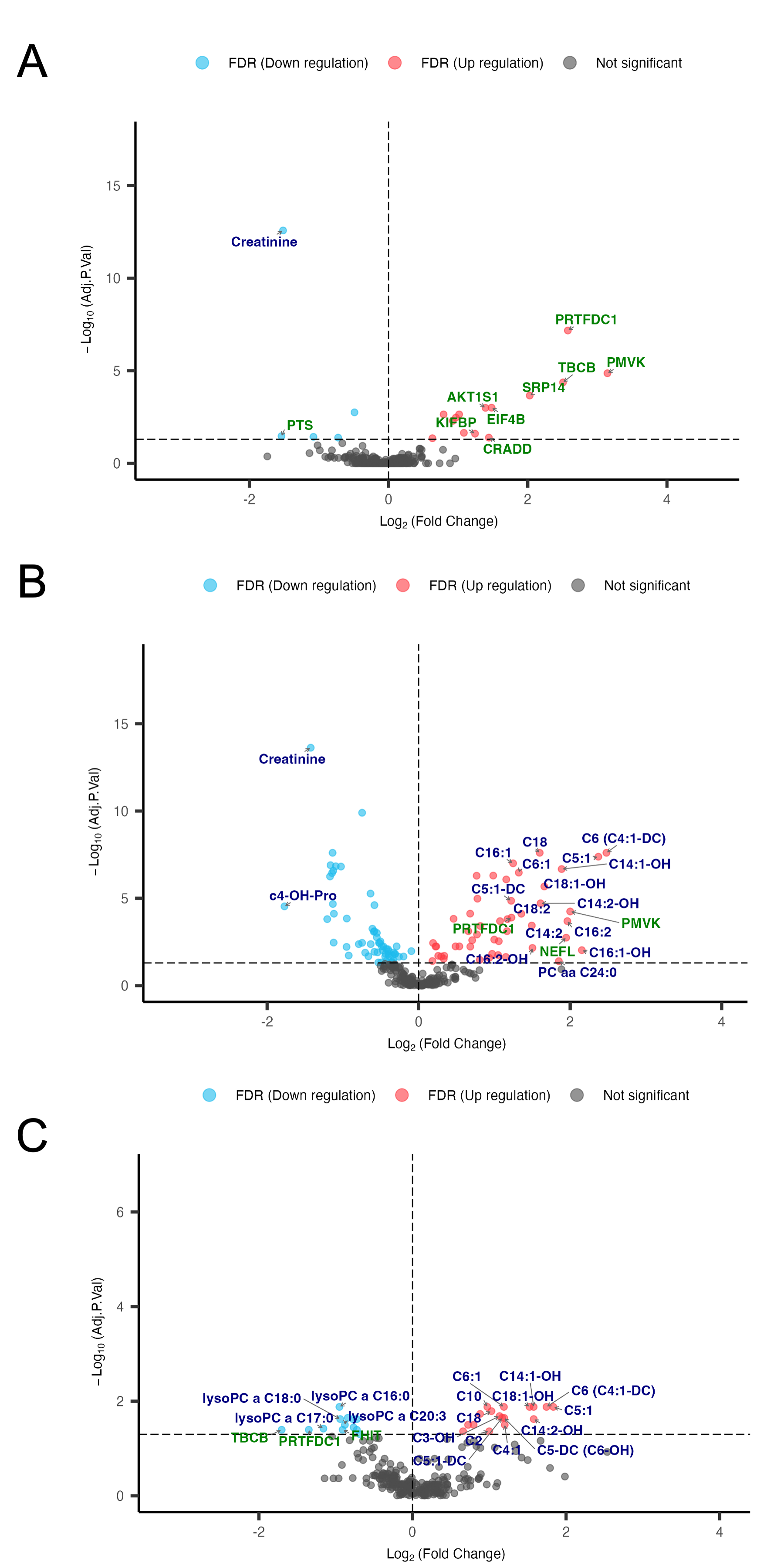


Figure S3. Volcano plot resulting from differential analysis using plasma samples between A. SMN2(Copy2) vs control. B. SMN2(Copy3/4) vs control.


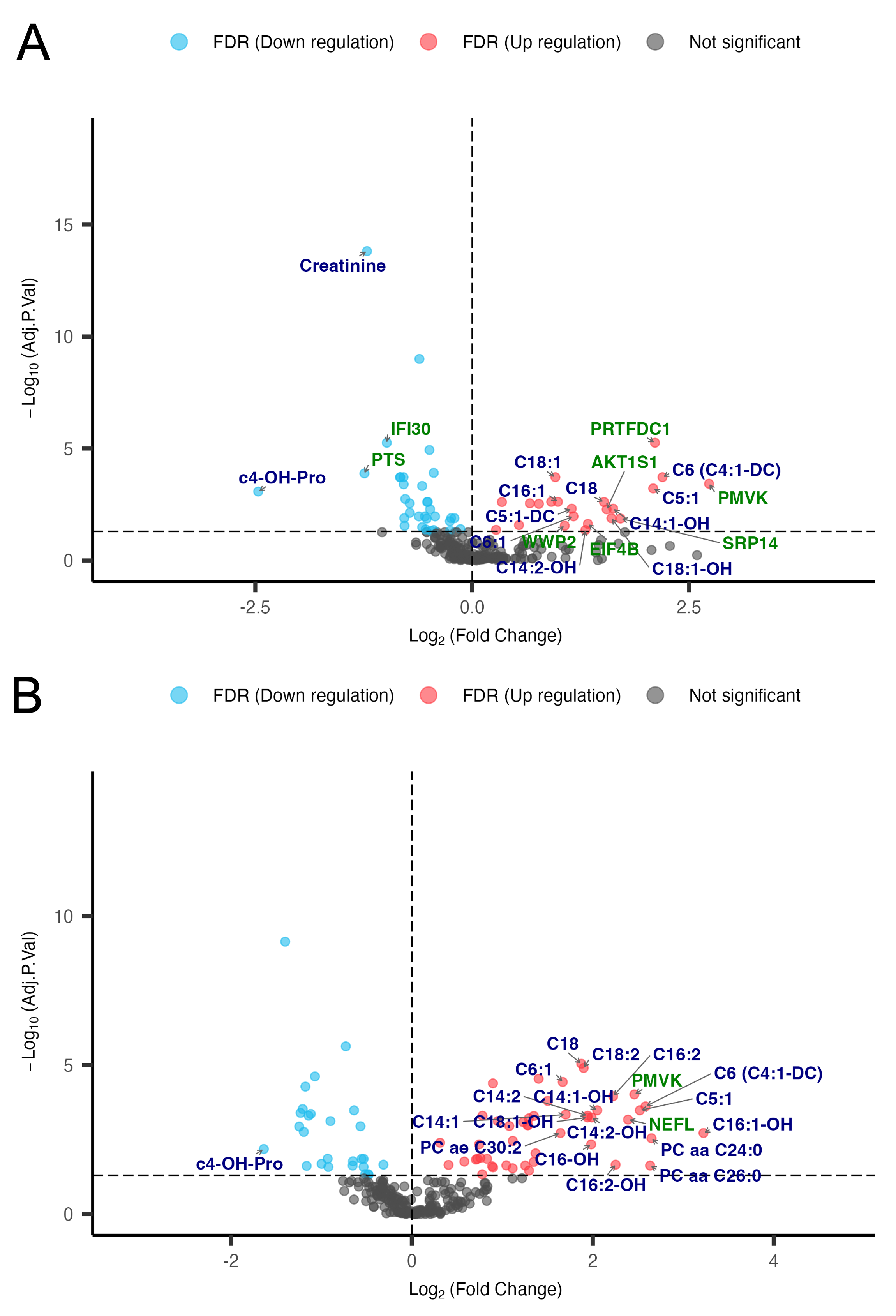


Figure S4. Top 5 altered analytes in each comparison using plasma. A. SMA(Baseline) vs control. B. SMA(J184) vs control. C. SMA(J184) vs SMA(Baseline). D. SMA(Mild) vs control. E. SMA(Severe) vs control. F. SMA(Severe) vs SMA(Mild). G. SMN2(Copy2) vs control. H. SMN2(Copy3/4) vs control.


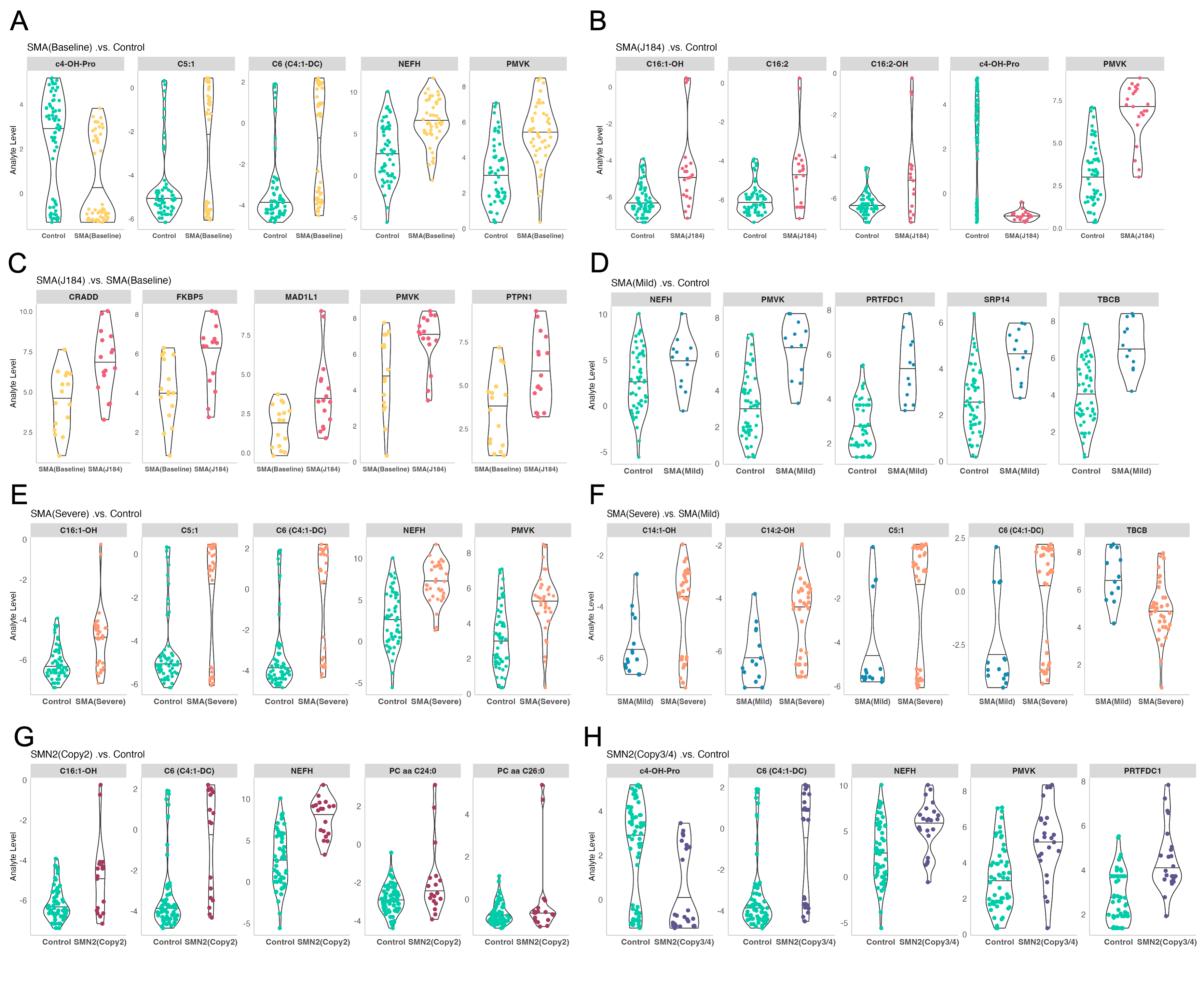


Figure S5. Volcano plot resulting from differential analysis using CSF samples between SMA(J184) vs control.


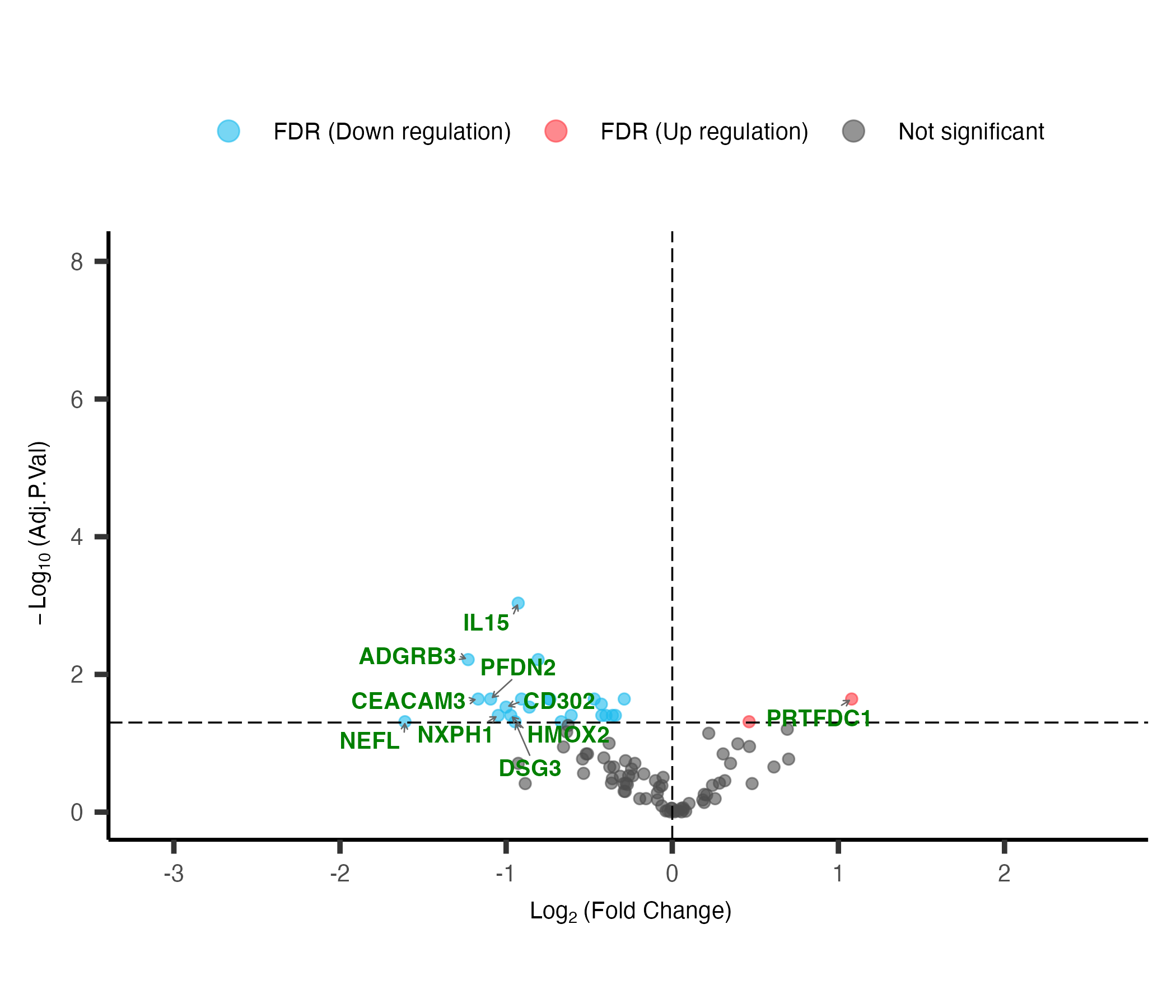

Supplement: Multimedia component 2 [file mmc2.docx]
